# OP68 Treatment Of Benign Prostatic Hyperplasia Among Remote Indigenous Populations: Experience With A Minimally Invasive Therapy In The Brazilian Amazon Rainforest

**DOI:** 10.1017/S0266462325101256

**Published:** 2025-12-29

**Authors:** Maiara Antonio, Isabela Horti, Henrique Tortele, Anna Pereira, Murilo Contó

## Abstract

**Introduction:**

Initial symptoms of benign prostatic hyperplasia (BPH) include difficulty urinating and low urinary flow. Worsening of BPH can lead to acute urinary retention and renal failure. Although it is fully treatable in urban centers, indigenous and remote area people are severely impacted. The experience presented herein shows how minimally invasive treatments can promote access to health care even in remote areas.

**Methods:**

In October 2024, after 48 hours of travel—two flights and more than six hours by boat—a team of six people in association with a specialized non-governmental organization arrived at the community of Assunção do Içana, in the middle of the Brazilian Amazon rainforest. Screening of the community population with BPH was initiated a year earlier and eligibility was double-checked with the lower urinary tract symptoms visual score and ultrasound. Patient interest in undergoing the procedure was a criterion. A mobile surgical center was set up in the community. Waterproof and thermally insulating materials were used to create a controlled environment, with adequate temperature and sterile conditions.

**Results:**

A general practitioner specialized in indigenous health initially screened 180 patients, and 14 procedures were performed by a urologist. The mean room time (from patient entry to discharge) and time from anesthesia to the placement of the catheter were 55 minutes and 29 minutes, respectively. The mean urologist’s field time was 20 minutes, while mean time for use of the Rezūm water vapor therapy disposable delivery device was four minutes. Challenges faced during the expedition were power outages, lack of full leg braces (making patient positioning more difficult), and an error in oxygen delivery. All issues were quickly resolved and did not affect patient care.

**Conclusions:**

In areas where it can take days to reach the nearest hospital, minimally invasive therapy that does not require hospitalization may be the only option. The Rezūm device has proven to be an effective and adaptable solution. Simplicity of the procedure and minimal need for resources were key to treatment success. This expedition highlighted the transformative potential of innovative technologies.

